# Associations of Domain‐Specific Physical Activity With Mental Health Symptoms Among Finnish Employed Adults: A Population‐Based Study

**DOI:** 10.1002/ejsc.70118

**Published:** 2026-01-07

**Authors:** Juuso J. Jussila, Anna Pulakka, Kaija Appelqvist‐Schmidlechner, Jaana I. Halonen, Jenni Ervasti, Paula Salo, Jouni Lahti, Santtu Mikkonen, Timo Lanki

**Affiliations:** ^1^ Institute of Public Health and Clinical Nutrition University of Eastern Finland Kuopio Finland; ^2^ Department of Public Health Finnish Institute for Health and Welfare (THL) Helsinki Finland; ^3^ Department of Healthcare and Social Welfare Finnish Institute for Health and Welfare (THL) Helsinki Finland; ^4^ Research Unit of Population Health University of Oulu Oulu Finland; ^5^ Division of Psychobiology and Epidemiology, Department of Psychology Stockholm University Stockholm Sweden; ^6^ Finnish Institute of Occupational Health Helsinki Finland; ^7^ Department of Psychology and Speech‐Language Pathology University of Turku Turku Finland; ^8^ Department of Public Health University of Helsinki Helsinki Finland; ^9^ Department of Environmental and Biological Sciences University of Eastern Finland Kuopio Finland

**Keywords:** active commuting, depressive symptoms, leisure‐time physical activity, occupational physical activity, psychological distress

## Abstract

Leisure‐time physical activity has consistently been associated with better mental health. However, evidence on active commuting and occupational physical activity is less conclusive. We examined cross‐sectional associations of domain‐specific physical activity with depressive symptoms and psychological distress among Finnish employed adults. We included 3439 adults (mean age 45.0 years; 51% female) from the FinHealth 2017 Study. Based on commuting, occupational and leisure‐time physical activity behaviour, participants were categorised as passive or active commuters; sedentary, lightly active or moderately/highly active workers; and sedentary, recreationally active or exercisers/athletes, respectively. Daily active commuting volumes were also assessed. Logistic regression was used to estimate odds ratios, with models adjusted for key covariates. High volumes of active commuting (≥ 30 min a day) were associated with higher odds of depressive symptoms (OR 1.58, 95% CI 1.18–2.13), whereas no associations were observed for lower active commuting volumes or when active commuting was analysed as a binary variable. Regarding occupational physical activity, lightly active workers were less likely to experience psychological distress (OR 0.62, 95% CI 0.40–0.97) compared to sedentary workers, whereas no associations were observed for moderately or highly active workers. Regarding leisure‐time physical activity, exercisers and athletes had lower odds of depressive symptoms (OR 0.44, 95% CI 0.32–0.61) and psychological distress (OR 0.34, 95% CI 0.21–0.55) compared to sedentary individuals, as did recreationally active adults. Leisure‐time and light occupational physical activity, but not active commuting, were associated with fewer mental health symptoms. Potential mental health benefits of physical activity may be domain‐ and volume‐specific.

## Highlights

- High volumes of active commuting were associated with more depressive symptoms, whereas light occupational physical activity was associated with less psychological distress.
- Engaging in recreational activities, exercise and sports was linked to better mental health.
- Mental health outcomes of physical activity may be domain‐ and volume‐specific and promoting leisure‐time physical activity might enhance mental health among employed adults.

## 1 Introduction

Engaging in regular leisure‐time physical activity can effectively reduce the risk of major mental disorders, such as depression and anxiety (Vella et al. 2023; White et al. 2017). Moreover, physical activity and exercise interventions have been consistently shown to alleviate a wide range of mental health symptoms in both healthy and clinical populations (Singh et al. 2023; Heissel et al. 2023; Recchia et al. 2022). As the worldwide prevalence and burden of depressive and anxiety disorders have surged in recent years (Santomauro et al. 2021), physical activity promotion could emerge as one of the main solutions for tackling this timely public health challenge. However, it remains unclear whether commuting‐ and work‐related physical activity can improve population mental health and how these domains differ from leisure‐time physical activity.

Promoting active commuting, that is, walking and cycling to work, has been recognised as a key strategy for increasing global physical activity levels, enhancing population health and mitigating climate change (World Health Organisation 2022). Previous studies have demonstrated that regular active commuting—particularly by cycling—is associated with a reduced risk of major noncommunicable diseases, such as cardiovascular disease and type 2 diabetes (Dinu et al. 2018; Wu et al. 2021), a lower risk of all‐cause and cardiovascular mortality (Dutheil et al. 2020) and reduced carbon emissions from motorised traffic (Brand et al. 2021). Nevertheless, evidence on its associations with mental health is mixed.

In a recent systematic review, five out of seven studies—comprising both cross‐sectional and longitudinal designs—did not find an association between active commuting and depression (Marques et al. 2020). Additionally, one of the longitudinal studies, which found that moderate (0.25–1.5 miles a day) and high (> 1.5 miles a day) volumes of walking were associated with a lower risk of depressive symptoms among older Japanese American men, did not examine active commuting per se but rather walking in general (Smith et al. 2010). In contrast, one longitudinal study from the United States found that individuals who shifted from exclusive car use to more active commuting reported fewer depressive symptoms compared to those who remained inactive (Knott et al. 2018).

In contrast to the systematic review, additional longitudinal evidence suggests potential mental health benefits of active commuting. For example, a UK study observed that increased active commuting was associated with improvements in general mental health compared to passive commuting (Jacob et al. 2020), and another British study found that cycle commuting was linked to a lower likelihood of antidepressant and/or anxiolytic prescriptions during a 5‐year follow‐up (Berrie et al. 2024). Few cross‐sectional studies further support these findings, with active commuting being associated with higher positive mental health among Finnish adults (Tamminen et al. 2020), lower stress levels among Spanish bicycle commuters (Avila‐Palencia et al. 2017) and a lower prevalence of depression among Brazilian adults reporting low volumes of active transportation (60–89 min a week) (Schuch et al. 2021). Overall, although some studies suggest beneficial associations between active commuting and mental health, the evidence remains mixed. This highlights the need for further research using more comprehensive and representative data to deepen our understanding of these associations.

Compared with leisure‐time physical activity, high levels of occupational physical activity have not been associated with similar benefits for physical health (Coenen et al. 2024; Cillekens et al. 2020; Holtermann et al. 2018). Physically strenuous work has also generally been linked to mental‐ill health (Vella et al. 2023; White et al. 2017; Cillekens et al. 2020). For example, recent studies from Korea and Brazil found that workers who engaged in work‐related physical activities reported more depressive symptoms than sedentary workers across various occupations (Joo et al. 2023; Werneck et al. 2023). Nonetheless, the evidence for occupational physical activity is even more limited than for active commuting (Vella et al. 2023; White et al. 2017).

The aim of this study was to address these evidence gaps by examining the associations of active commuting, occupational physical activity and—for comparison—leisure‐time physical activity with depressive symptoms and psychological distress among Finnish employed adults. Unlike most prior studies, we were able to investigate these associations in a representative cohort of adults using data from the FinHealth 2017 Study.

## 2 Materials and Methods

### 2.1 Study Design and Participants

The FinHealth 2017 Study (Borodulin and Sääksjärvi 2019) was conducted by the Finnish Institute for Health and Welfare from January 31 to May 17, 2017. The study aimed to produce reliable and up‐to‐date information on health, wellbeing and functional capacity of the Finnish adult population. The FinHealth 2017 Study data used in this observational cross‐sectional analytic study were collected by mailed surveys.

Sampling was conducted using a systematic probability proportional to size (PPS‐SYS) method, with mainland Finland divided into 50 strata based on national healthcare districts. Population sizes were obtained from the Population Register Centre and used to determine the sample sizes for each healthcare district. The exact population sizes, along with the actual samples, were then obtained from the Population Register Centre at the time of the sampling to calibrate the weights for analyses. The sample size of individuals in each stratum was proportional to the corresponding population size (Borodulin and Sääksjärvi 2019).

Overall, 10,305 adults (≥ 18‐years‐old) living in Finland were invited to participate in the FinHealth 2017 Study. Of the eligible 10,247 individuals (alive and with known address information), 7046 participated in at least the self‐administered survey phase of the data collection (69% participation rate). We excluded participants who were unemployed, working from home or over 74‐years‐old (*N* = 3287). To be able to run complete case analyses, those with missing physical activity, mental health or main covariate data were also removed (*N* = 320). The final analytical sample comprised 3439 adults. Notably, the number of participants aged 65–74 years in the analytical sample was low (*N* = 132), as the typical retirement age in Finland is 65.

### 2.2 Active Commuting, Leisure‐Time Physical Activity and Occupational Physical Activity

Active commuting was self‐reported in response to the question: ‘On your way to work or school, how many minutes do you travel by walking, cycling, or using other active transportation modes? Add up the journeys to and from work or school’ (Borodulin and Sääksjärvi 2019). The response options were ‘I only use motorised vehicles for commuting’, ‘less than 15 min’, ‘15–29 min’, ‘30–60 min’ and ‘more than 60 min’ a day. Based on responses, we categorised the participants as passive (exclusive use of motorised vehicles) or active commuters (any amount of daily active commuting).

Occupational physical activity profile was self‐reported in response to the question: ‘How demanding is your work physically? Please choose the option that best applies to your situation’ (Borodulin and Sääksjärvi 2019). Based on responses, we initially categorised participants as sedentary (‘My work is mainly done sitting down and I do not walk much during my working hours’), lightly active (‘I walk quite a lot in my work, but I do not have to lift or carry heavy objects’), moderately active (‘I have to walk and lift a lot or take the stairs or go uphill’) and highly active (‘My work is heavy manual labour in which I have to lift or carry heavy objects, dig, shovel, or chop, etc.’) groups. Subsequently, the moderately and highly active workers were combined into one group due to a low number of highly active workers (*N* = 287).

Based on the Saltin–Grimby Physical Activity Level Scale (SGPALS), which has demonstrated acceptable concurrent and predictive validity in various cohorts over the past decades (Grimby et al. 2015), leisure‐time physical activity profile was self‐reported in response to the question: ‘How much do you exercise and exert yourself physically in your leisure‐time?’ (Borodulin and Sääksjärvi 2019). Based on responses, we initially categorised participants as sedentary (‘I read, watch television, and do other activities involving minimal physical movement or exertion’), recreationally active (‘I walk, cycle, and engage in other activities for several hours a week, such as fishing, hunting, or light home gardening’), exercisers (‘I exercise for several hours a week, including running, jogging, cross‐country skiing, fitness training, swimming, ball games, or strenuous garden work’) or athletes (‘I practice strenuous sports several times per week, including competitive sports such as running, orienteering, cross‐country skiing, swimming, and ball games’). Subsequently, the exercisers and athletes were combined into one group due to a low number of athletes (*N* = 90).

Except for the SGPALS, the physical activity measures used in this study have not been formally validated. However, they have been widely used in prior nationally representative cohort studies (e.g., the National FINRISK Study) (Borodulin and Sääksjärvi 2019). These measures were retained in the FinHealth 2017 Study to ensure comparability with earlier studies and to enable long‐term monitoring of physical activity trends among Finnish adults. All measures were also pilot‐tested in a randomised sample of Finnish adults (*N* = 100) to ensure their feasibility.

### 2.3 Depressive Symptoms and Psychological Distress

Depressive symptoms were measured with a question from the Structured Clinical Interview for DSM‐IV Axis I Disorders (SCID‐I) (First et al. 1997). It utilised two dichotomous items (yes/no): ‘Over the last 12 months, have you had a period of at least 2 weeks when you (1) felt down, low‐spirited, or depressed, and (2) lost interest in most things, such as hobbies, work, or other activities that usually give you pleasure?’. Participants who answered ‘yes’ to either of the items were categorised as having depressive symptoms.

Psychological distress was measured with the validated Mental Health Inventory‐5 (MHI‐5) (McHorney and Ware 1995). The MHI‐5 comprised five items: ‘How much of the time in the past 4 weeks have you (1) been a very nervous person, (2) felt so down in the dumps that nothing could cheer you up, (3) felt calm and peaceful, (4) felt downhearted and blue, and (5) been a happy person?’. The response options were all of the time (scored as 1), most of the time (scored as 2), a good bit of the time (scored as 3), some of the time (scored as 4), a little of the time (scored as 5) and none of the time (scored as 6). The scoring was done in reverse for the Questions 3 and 5 as they represent positive feelings. The raw scores (ranging from 5 to 30) were then converted to a 0–100‐point scale, with low scores indicating more psychological distress. Although there is not one established cut‐off point for identifying clinically significant psychological distress, the commonly used cut‐off, a total of 52 points or below, was applied (Borodulin and Sääksjärvi 2019).

### 2.4 Covariates

Guided by the literature (Bauman et al. 2012; Mrazek and Haggerty 1994) and data availability (Bowler et al. 2010), we identified and included key covariates associated with physical activity behaviour and mental health: age, sex, education level, household income (an income of ≤ 25,000 € per year was defined as low income, 25,001€–80,000€ per year as middle income, and > 80,000 € per year as high income), marital status, children (living at home), smoking status and hazardous or problem drinking (measured by the abbreviated Alcohol Use Disorder Identification Test) (Bush et al. 1998). In sensitivity analyses, we also controlled for body mass index, adequate sleep, self‐rated health and history of chronic diseases (any of the following diseases: hypertension, heart failure, coronary heart disease, myocardial infarction, cerebrovascular disease, type 1 diabetes, type 2 diabetes, cancer, rheumatoid arthritis, chronic kidney disease, degenerative disc disease, asthma, chronic obstructive pulmonary disease and sleep apnoea) as they can both confound and mediate the studied associations. All covariates were self‐reported.

### 2.5 Statistical Analyses

We investigated the distribution of study population characteristics (i.e., all covariates) across commuting, leisure‐time physical activity, occupational physical activity, depressive symptoms and psychological distress groups. Tests of differences across groups were conducted by the chi‐square test (χ^2^).

As our main analysis, we used logistic regression models to investigate associations of active commuting, leisure‐time physical activity and occupational physical activity with depressive symptoms and psychological distress. The models were first adjusted for age and sex. Second, they were adjusted for other sociodemographic factors (education level, household income, marital status and children living at home) and health behaviours (smoking status and hazardous or problem drinking). Third, in the main model, we additionally adjusted for other physical activity domains.

In additional analyses, we used a four‐level active commuting variable to examine the relationships between different volumes of active commuting and mental health symptoms: passive commuting (*N* = 1819), < 15 min (*N* = 459), 15–29 min (*N* = 634), and ≥ 30 min (*N* = 527) a day. We combined the ‘30–60 min a day’ and ‘more than 60 min a day’ groups due to a low number of participants in the latter (*N* = 162). Furthermore, we tested for interactions by age group and sex in the main regression model. As the only statistically significant interaction emerged between age group and active commuting volume in relation to depressive symptoms, we performed a stratified analysis to explore this association further.

In sensitivity analyses, we individually included body mass index, adequate sleep, self‐rated health and history of main chronic diseases into the main regression model. We also tested an alternative main model in which we replaced household income with income satisfaction.

Passive commuters were used as a reference group for all regression analyses on active commuting, sedentary individuals for leisure‐time physical activity and sedentary workers for occupational physical activity. The absence of multicollinearity was confirmed with variance inflation factors. Survey weights were used to adjust for nonparticipation using the *R* package survey, a method commonly applied in large population‐based studies (Coenen et al. 2024). Detailed information on the register variables used in the weighting model can be found in the FinHealth 2017 Study methods report (Borodulin and Sääksjärvi 2019).

All analyses were performed using *R* (4.3.0) and RStudio (2023.09.0) software. Results from the logistic regression analyses are presented as odds ratios (OR) with 95% confidence intervals (CI).

## 3 Results

### 3.1 Descriptive Characteristics

Of all participants (mean age 45.0 years; 51% female), 47% were categorised as active commuters, whereas 53% were passive commuters (Table 1). Active commuters were more often female than male, unmarried than married, nonsmokers than smokers and those with higher education, no children living at home, better health status and lower levels of occupational physical activity. Active commuting was more common among exercisers and athletes than recreationally active adults and those with a sedentary lifestyle.

**TABLE 1:** Characteristics of the analytical sample by a commuting profile. Values are numbers (percentages).

Characteristics	Passive commuters *N* = 1819	Active commuters *N* = 1620
Sociodemographic		
Age		
18–34‐years‐old	381 (20.9)	476 (29.4)
35–54‐years old	1016 (55.9)	712 (43.9)
55–74‐years old	422 (23.2)	432 (26.7)
Sex		
Female	833 (45.8)	937 (57.8)
Male	986 (54.2)	683 (42.2)
Education level		
Primary education	145 (8.0)	130 (8.0)
Secondary education	724 (39.8)	511 (32.5)
Higher education	950 (52.2)	979 (60.4)
Household income		
Low income	143 (7.9)	333 (20.5)
Middle income	1248 (68.6)	957 (59.1)
High income	428 (23.5)	330 (20.4)
Income satisfaction		
Unsatisfied	180 (10.0)	206 (12.7)
Satisfied	1638 (90.0)	1412 (87.3)
Marital status		
Unmarried, divorced, or widowed	368 (20.2)	520 (32.1)
Married or cohabiting	1451 (79.8)	1100 (67.9)
Children (living at home)		
No	1000 (55.0)	1112 (68.6)
Yes, at least one	819 (45.0)	508 (32.4)
Health status		
Body mass index		
Normal weight or underweight	691 (38.0)	779 (48.1)
Overweight or obese	1128 (62.0)	841 (51.9)
Self‐rated health		
Poor or quite poor	48 (2.6)	56 (3.5)
Average	410 (22.6)	280 (17.3)
Good or quite good	1359 (74.8)	1278 (79.2)
Chronic diseases		
No	846 (48.3)	829 (53.2)
Yes, at least one	907 (51.7)	729 (46.8)
Health behaviour		
Smoking status		
Nonsmoker	958 (52.7)	1026 (63.3)
Former smoker	471 (25.9)	361 (22.3)
Smoker	390 (21.4)	233 (14.4)
Hazardous or problem drinking		
No	1310 (72.0)	1217 (75.1)
Yes	509 (28.0)	403 (24.9)
Adequate sleep		
No, seldom or never or cannot tell	462 (25.6)	395 (24.5)
Yes, almost always or often	1346 (74.4)	1219 (75.5)
Physical activity		
Leisure‐time physical activity		
Sedentary	420 (23.1)	354 (21.8)
Recreationally active	787 (43.3)	667 (41.2)
Exercisers and athletes	612 (33.6)	599 (37.0)
Occupational physical activity		
Sedentary	677 (37.2)	790 (48.8)
Lightly active	444 (24.4)	411 (25.4)
Moderately or highly active	698 (38.4)	419 (25.8)

*Note:* All variables are globally significantly different between commuting groups at *p* < 0.05, except adequate sleep (0.467) and leisure‐time physical activity (0.124).

For occupational physical activity, 32% of participants were moderately or highly active workers, 25% were lightly active workers and 43% were sedentary workers (Table 2). Compared to sedentary workers, moderately or highly active workers were more often male than female, less educated and from lower‐income households. Although there were no clear differences in health status between the groups, moderately and highly active workers generally had poorer health behaviours, such as less physical activity in other domains, along with higher rates of smoking and hazardous or problem drinking. Compared to lightly active workers, sedentary workers were more physically active in their leisure time and had a better socioeconomic status.

**TABLE 2:** Characteristics of the analytical sample by the occupational physical activity profile. Values are numbers (percentages).

Characteristics	Sedentary *N* = 1467	Lightly active *N* = 855	Moderately or highly active *N* = 1117
Sociodemographic			
Age			
18–34‐years‐old	380 (25.9)	183 (21.4)	294 (26.3)
35–54‐years old	757 (51.6)	418 (48.9)	553 (49.5)
55–74‐years old	330 (22.5)	254 (29.7)	270 (24.2)
Sex			
Female	803 (54.7)	491 (57.4)	476 (42.6)
Male	664 (45.3)	364 (42.6)	641 (57.4)
Education level			
Primary education	77 (5.3)	62 (7.2)	136 (12.2)
Secondary education	292 (19.9)	294 (34.4)	649 (58.1)
Higher education	1098 (74.8)	499 (58.4)	332 (29.7)
Household income			
Low income	184 (12.5)	128 (15.0)	164 (14.7)
Middle income	804 (54.8)	558 (65.2)	843 (75.5)
High income	479 (32.7)	169 (19.8)	110 (9.8)
Income satisfaction			
Unsatisfied	161 (11.0)	81 (9.5)	144 (12.9)
Satisfied	1305 (89.0)	773 (90.5)	972 (87.1)
Marital status			
Unmarried, divorced or widowed	372 (25.4)	225 (26.3)	291 (26.1)
Married or cohabiting	1095 (74.6)	630 (73.7)	826 (73.9)
Children (living at home)			
No	874 (59.6)	557 (65.1)	681 (61.0)
Yes, at least one	593 (40.4)	298 (34.9)	436 (39.0)
Health status			
Body mass index			
Normal weight or underweight	652 (44.4)	365 (42.7)	453 (40.6)
Overweight or obese	815 (55.6)	490 (57.3)	664 (59.4)
Self‐rated health			
Poor or quite poor	36 (2.5)	27 (3.2)	41 (3.7)
Average	264 (18.0)	152 (17.8)	274 (24.6)
Good or quite good	1165 (79.5)	673 (79.0)	799 (71.7)
Chronic diseases			
No	722 (51.2)	425 (51.6)	528 (49.0)
Yes, at least one	689 (48.8)	398 (48.4)	549 (51.0)
Health behaviour			
Smoking status			
Nonsmoker	947 (64.6)	519 (60.7)	518 (46.4)
Former smoker	330 (22.5)	197 (23.0)	305 (27.3)
Smoker	190 (12.9)	139 (16.3)	294 (26.3)
Hazardous or problem drinking			
No	1113 (75.9)	651 (76.1)	753 (68.3)
Yes	354 (24.1)	204 (23.9)	354 (32.7)
Adequate sleep			
No, seldom or never or cannot tell	377 (25.8)	202 (23.8)	278 (25.0)
Yes, almost always or often	1083 (74.2)	648 (76.2)	834 (75.0)
Physical activity			
Active commuting			
Passive commuters	677 (46.1)	444 (51.9)	698 (62.5)
Active commuters	790 (53.9)	411 (48.1)	419 (37.5)
Leisure‐time physical activity			
Sedentary	321 (21.9)	177 (20.7)	276 (24.7)
Recreationally active	557 (38.0)	391 (45.7)	506 (45.3)
Exercisers and athletes	589 (40.1)	287 (33.6)	335 (30.0)

*Note:* All variables are globally significantly different between occupational physical activity groups at *p* < 0.05, except marital status (0.859), body mass index (0.141), adequate sleep (0.545), chronic diseases (0.448) and income satisfaction (0.054).

For leisure‐time physical activity, 35% of participants were categorised as exercisers and athletes, 42% as recreationally active and 23% as sedentary (Table 3). Compared to sedentary and recreationally active adults, exercisers and athletes were more likely to be younger males with higher socioeconomic status and self‐rated health. They also had fewer chronic diseases and lower levels of occupational physical activity and were less likely to be overweight or smokers. Differences between recreationally active and sedentary groups were less prominent, although also recreationally active individuals tended to have a better socioeconomic status, self‐rated health and health behaviours as well as lower body mass index.

**TABLE 3:** Characteristics of the analytical sample by the leisure‐time physical activity profile. Values are numbers (percentages).

Characteristics	Sedentary *N* = 774	Recreationally active *N* = 1454	Exercisers and athletes *N* = 1211
Sociodemographic			
Age			
18–34‐years‐old	184 (23.8)	261 (18.0)	412 (34.0)
35–54‐years old	386 (49.9)	745 (51.2)	597 (49.3)
55–74‐years old	204 (26.3)	448 (30.8)	202 (16.7)
Sex			
Female	402 (51.9)	798 (54.9)	570 (47.1)
Male	372 (48.1)	656 (45.1)	641 (52.9)
Education level			
Primary education	94 (12.1)	136 (9.4)	45 (3.7)
Secondary education	307 (39.7)	550 (37.8)	378 (31.2)
Higher education	373 (48.1)	768 (52.8)	788 (65.1)
Household income			
Low income	148 (19.1)	198 (13.6)	130 (10.7)
Middle income	514 (66.4)	963 (66.2)	728 (60.1)
High income	112 (14.5)	728 (20.2)	353 (29.2)
Income satisfaction			
Unsatisfied	131 (17.0)	151 (10.4)	104 (8.6)
Satisfied	641 (83.0)	1302 (89.6)	1107 (91.4)
Marital status			
Unmarried, divorced or widowed	231 (29.8)	331 (22.8)	326 (26.9)
Married or cohabiting	543 (70.2)	1123 (77.2)	885 (73.1)
Children (living at home)			
No	494 (63.8)	917 (63.1)	701 (57.9)
Yes, at least one	280 (36.2)	537 (36.9)	510 (42.1)
Health status			
Body mass index			
Normal weight or underweight	251 (32.4)	608 (41.8)	611 (50.5)
Overweight or obese	523 (67.6)	846 (58.2)	600 (49.5)
Self‐rated health			
Poor or quite poor	55 (7.1)	39 (2.7)	10 (0.8)
Average	277 (35.8)	327 (22.6)	86 (7.1)
Good or quite good	441 (57.1)	1081 (74.7)	1115 (92.1)
Chronic diseases			
No	348 (46.4)	654 (47.0)	673 (57.6)
Yes, at least one	402 (53.6)	738 (53.0)	496 (42.4)
Health behaviour			
Smoking status			
Nonsmoker	388 (50.1)	801 (55.1)	795 (65.6)
Former smoker	182 (23.5)	354 (24.3)	296 (24.4)
Smoker	204 (26.4)	299 (20.6)	120 (10.0)
Hazardous or problem drinking			
No	527 (68.1)	1096 (75.4)	904 (74.6)
Yes	247 (31.9)	358 (24.6)	307 (25.4)
Adequate sleep			
No, seldom or never or cannot tell	248 (32.2)	346 (23.9)	263 (21.8)
Yes, almost always or often	521 (67.8)	1102 (76.1)	942 (78.2)
Physical activity			
Active commuting			
Passive commuters	420 (54.3)	787 (54.1)	612 (50.5)
Active commuters	354 (45.7)	667 (45.9)	599 (49.5)
Occupational physical activity			
Sedentary	321 (41.5)	557 (38.3)	589 (48.6)
Lightly active	177 (22.9)	391 (26.9)	287 (23.7)
Moderately or highly active	276 (35.6)	506 (34.8)	335 (27.7)

*Note:* All variables are globally significantly different between leisure‐time physical activity groups at *p* < 0.05, except active commuting (0.124).

For mental health, 22% of participants reported at least a 2‐week period of depressive symptoms over the past 12 months, whereas 7% reported psychological distress during the past 4 weeks (Supporting Information S1: Table S1). Prevalence of both types of mental health problems was higher among females than males, unmarried than married/cohabiters and those with low household income, no children living at home, poorer self‐rated health, chronic diseases and detrimental health behaviours, such as smoking and hazardous or problem drinking.

### 3.2 Active Commuting and Mental Health

**FIGURE 1:**
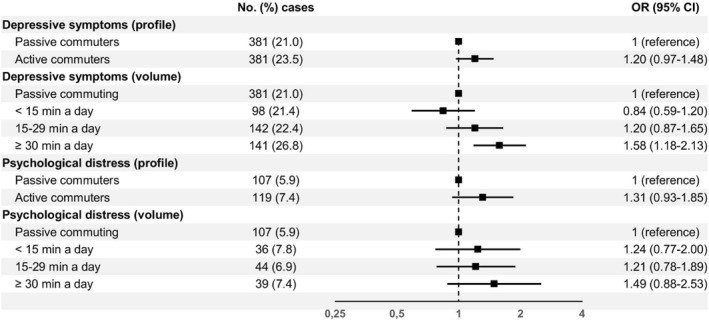
Adjusted odds ratios with 95% confidence intervals for mental health symptoms by commuting profile and active commuting volume. Adjusted for age, sex, education level, household income, marital status, children (living at home), smoking status, hazardous or problem drinking, occupational physical activity and leisure‐time physical activity.

In the main regression model, we did not observe associations between active commuting and depressive symptoms or psychological distress when using the binary commuting variable (Figure 1). However, when we used the four‐level variable, we found that adults who walked or cycled to work for at least 30 min a day had 58% higher odds of depressive symptoms compared to those who commuted exclusively by motorised transportation (OR 1.58, 95% CI 1.18–2.13). Stratified analyses showed that this association emerged only among participants aged 18–34 years (OR 2.64, 95% CI 1.42–4.94) and 55–74 years (OR 1.60, 95% CI 1.01–2.56). No significant associations were observed for low (< 15 min a day) or moderate (15–29 min a day) volumes of active commuting.

Compared to the crude models (adjusted only for age and sex), adjusting for sociodemographic factors and health behaviours slightly attenuated most associations, whereas controlling for other physical activity domains did not have a large effect on the findings (Supporting Information S1: Table S2). In sensitivity analyses, we also found that active commuters had higher odds of depressive symptoms (OR 1.28, 95% CI 1.03–1.58) and psychological distress (OR 1.43, 95% CI 1.03–1.97) when household income was replaced with income satisfaction.

### 3.3 Occupational Physical Activity and Mental Health

**FIGURE 2:**
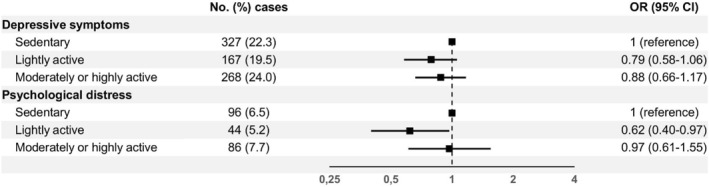
Adjusted odds ratios with 95% confidence intervals for mental health symptoms by occupational physical activity profile. Adjusted for age, sex, education level, household income, marital status, children (living at home), smoking status, hazardous or problem drinking, active commuting and leisure‐time physical activity.

In the main regression model, we observed that lightly active workers had 38% lower odds of psychological distress compared to sedentary workers (OR 0.62, 95% CI 0.40–0.97) (Figure 2). No statistically significant associations were found for depressive symptoms or for moderately or highly active workers.

Compared to the crude models, adjusting for other sociodemographic factors and health behaviours attenuated all other associations except the one between lightly active work and psychological distress (Supporting Information S1: Table S3). In sensitivity analyses, adjusting for self‐rated health and history of chronic diseases, as well as replacing household income with income satisfaction, also attenuated the association between lightly active work and psychological distress to null.

### 3.4 Leisure‐Time Physical Activity and Mental Health

**FIGURE 3:**
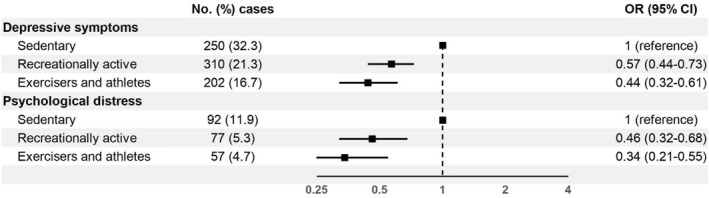
Adjusted odds ratios with 95% confidence intervals for mental health symptoms by leisure‐time physical activity profile. Adjusted for age, sex, education level, household income, marital status, children (living at home), smoking status, hazardous or problem drinking, active commuting and leisure‐time physical activity.

In the main regression model, we observed that exercisers and athletes had 56% (OR 0.44, 95% CI 0.32–0.61) and 66% (OR 0.34, 95% CI 0.21–0.55) lower odds of depressive symptoms and psychological distress, respectively, compared to sedentary adults (Figure 3). Similarly, recreationally active adults had 43% lower odds of depressive symptoms (OR 0.57, 95% CI 0.44–0.73) and 54% lower odds of psychological distress (OR 0.46, 95% CI 0.32–0.68) than those in the sedentary group.

Compared to the crude models, the associations attenuated slightly after adjusting for sociodemographic factors and health behaviours, but not after controlling for other physical activity domains (Supporting Information S1: Table S4). In sensitivity analyses, adjusting for self‐rated health had the largest effect on the findings. For example, although the other associations remained statistically significant, the association of psychological distress attenuated to null for exercisers and athletes (OR 0.63, 95% CI 0.36–1.08).

## 4 Discussion

We studied the associations of active commuting, occupational physical activity and leisure‐time physical activity with depressive symptoms over the past 12 months and psychological distress over the past 4 weeks among Finnish employed adults. Although active commuting was not associated with mental health symptoms when comparing all active commuters to passive commuters, we observed that high volumes of walking or cycling (≥ 30 min a day) were associated with higher odds of depressive symptoms, particularly among young and older employed adults. Conversely, lightly active workers were less likely to experience psychological distress than sedentary workers. Additionally, we found that participants who engaged in regular recreational physical activity or exercise/sports in their leisure‐time had lower odds of both depressive symptoms and psychological distress compared to sedentary individuals.

Although some studies have reported positive associations between active commuting and mental health (Knott et al. 2018; Jacob et al. 2020; Berrie et al. 2024; Tamminen et al. 2020; Avila‐Palencia et al. 2017), our main observation aligns with most prior findings that are contrary (Marques et al. 2020), including other evidence from Finland. For example, a longitudinal study demonstrated that an increase in walking or cycling to work over a 2‐year period was not associated with changes in psychological distress among Finnish public sector employees (Haukka et al. 2023). Additionally, commuting physical activity was not linked to positive mental health among young Finnish men (Appelqvist‐Schmidlechner et al. 2020). However, we were unable to differentiate between commuting in natural and urban environments, a factor that might moderate this association. For example, evidence from Spain, the Netherlands, Lithuania and the United Kingdom suggests that only active commuting through natural environments may provide mental health benefits (Zijlema et al. 2018). It is plausible that exposure to green or blue spaces during commuting fosters greater restoration and mood improvement compared to commuting in urban areas (Bowler et al. 2010).

Interestingly, similar to our previous work on adolescents (Jussila et al. 2022), we also observed that high volumes of active commuting were associated with a greater likelihood of depressive symptoms among young and older employed adults. Prior research suggests that long commutes—regardless of the travel mode—may be detrimental to health. For example, a Swedish study demonstrated that adults with longer commuting distances were more likely to be physically inactive, overweight and experience sleep problems (Raza et al. 2021). Nevertheless, these negative health outcomes do not explain our results that were unaffected by leisure‐time physical activity, body mass index and adequate sleep adjustments. Therefore, motivational determinants could offer an alternative explanation for our observation. For example, active commuting may not often be a chosen activity (Vella et al. 2023). Hence, like other behaviours driven by controlled motivation, high volumes of frequent, involuntary walking or cycling might become an everyday mental burden and predispose commuters to depressive symptoms (Stutzer and Frey 2008).

This theory is supported by a study from Belgium, which found that active commuting was associated with increased stress among blue‐collar workers but not among white‐collar workers (Asztalos et al. 2009). Blue‐collar workers may be more likely to walk or cycle due to financial constraints, whereas white‐collar commuters may autonomously choose these modes despite having access to other options, such as private cars. As young adults generally have lower incomes, this might also explain why the association was particularly strong in that subgroup. Nevertheless, we lacked information on commuting motives, which could have provided additional insights into the plausibility of this theory. Furthermore, adjusting for socioeconomic status indicators and occupational physical activity did not alter our findings, challenging this interpretation.

As for older adults, this somewhat unexpected association might be partially explained by age‐related decline in physical fitness and function, which could make longer durations of active commuting excessively demanding and, in turn, a source of stress.

Although high levels of occupational physical activity have generally been linked to adverse health outcomes, including mental ill‐health (Vella et al. 2023; White et al. 2017; Cillekens et al. 2020; Joo et al. 2023; Werneck et al. 2023), we found no associations between being a moderately or highly active worker and mental health symptoms. Instead, engaging in lightly active work was associated with less psychological distress compared to sedentary work. Although this study lacked detailed data on the participants' professions, the sedentary workers were the group with highest household incomes in our cohort. This may suggest that sedentary work represents professions with high job demands (e.g., competitive pressure), which are associated with an increased risk of work‐related stress and burnout (Bunjak et al. 2023). It is possible that this, rather than occupational physical activity level, explains why lightly active workers—potentially in less demanding jobs—reported less psychological distress.

Our results for leisure‐time physical activity are supported by a large body of prospective and experimental evidence (Vella et al. 2023; White et al. 2017; Singh et al. 2023; Heissel et al. 2023; Recchia et al. 2022). Again, motivational but also psychosocial factors could explain why recreational physical activity and exercise/sports appear to be more beneficial for mental health than active commuting and occupational physical activity. For example, participation in leisure‐time physical activity is autonomously motivated and often preceded by positive affective experiences (Vella et al. 2023), such as enjoyment, which are associated with better mental wellbeing—at least among adolescents (White et al. 2018; Doré et al. 2022). Moreover, leisure‐time physical activity can offer more opportunities for mastery experiences and social connectedness, contributing to greater improvements in self‐efficacy, feelings of belonging and, consequently, mental health (Vella et al. 2023; Nguyen Ho et al. 2023).

Neurobiological explanatory pathways are also plausible due to intensity difference between leisure‐time physical activity—particularly exercise and sports—and active commuting and occupational physical activity. For example, high‐intensity exercise can more efficiently stimulate the production of ‘feel‐good’ hormones, including serotonin, norepinephrine, dopamine and endorphins, compared to lower intensity physical activity, such as walking to or at work (Lin and Kuo 2013; Dishman and O’Connor 2009). In addition to mood improvements, these neurotransmitters play a role in various depressive symptoms, such as motivation and fatigue (Lin and Kuo 2013; Dishman and O’Connor 2009). Furthermore, higher exercise intensities are linked to increased brain‐derived neurotrophic factor levels, which could prevent or alleviate depressive symptoms via promoting hippocampal neurogenesis (Knaepen et al. 2010; Mandolesi et al. 2018). Nevertheless, we were unable to distinguish between walking and cycling, prohibiting the comparison with active commuting modes of different intensities.

The strengths of this study include a large, nationally representative cohort and a population‐based design, which enhances the generalisability of our observations at least to the broader Nordic employed adult population that shares similar physical activity behaviours. We were also able to perform comprehensive multivariable analyses, reducing residual confounding bias. In addition, misclassification of active commuting due to e‐bikes or e‐scooters is unlikely, as the data were collected in 2017, when e‐bikes accounted for less than 5% of all bikes in Finland and rentable e‐scooters had not yet been introduced in the country.

The main limitations of this study include the use of nonvalidated measures for commuting and occupational physical activity. However, all measurements of the FinHealth 2017 Study faced pilot tests prior to data collection, which should reduce the risk of measurement errors. The physical activity measures have also been successfully used in prior population‐based studies, such as the National FINRISK Study (Borodulin and Sääksjärvi 2019), and were retained to ensure comparability with these earlier surveys. Additionally, physical activity was self‐reported, potentially leading to overestimated walking or cycling frequencies and occupational physical demands (Lee et al. 2011). Nevertheless, both commuting and work can be considered a somewhat consistent, routine‐like behaviour, and we focused on leisure‐time and occupational physical activity profiles rather than specific volumes and intensities, which should reduce the likelihood of misclassification bias. Contrarily, the commuting question did not distinguish between walking or cycling as a primary or secondary mode of travel (e.g., as part of public transportation), which may have led to misinterpretation and attenuated the odds ratios, particularly at lower volumes of active commuting. Furthermore, our observations cannot be generalised to unemployed adults, who are more likely to be physically inactive and affected by mental health problems than the employed population (Honkonen et al. 2007).

Finally, no conclusions about the direction of the associations can be drawn due to the cross‐sectional design. As the relationship between physical activity and mental health is likely bidirectional (Azevedo Da Silva et al. 2012), the results regarding depressive symptoms over the past 12 months should specifically be interpreted with caution due to the longer time frame. Findings from randomised controlled trials support our observations for potential benefits of leisure‐time physical activity on depression and psychological distress, nonetheless (Singh et al. 2023; Heissel et al. 2023; Recchia et al. 2022).

## 5 Conclusion

This study highlights the importance of leisure‐time physical activity for population mental health, suggesting that this domain should be further emphasised in future physical activity guidelines and exercise prescriptions aimed at promoting mental wellbeing. Longitudinal and experimental studies across different motivational, cultural, geographical and climatic environments are required to further evaluate our findings on active commuting. Regarding occupational physical activity and mental health, future research should aim to compare different professions and occupational characteristics to provide deeper insights into these associations.

## Author Contributions

J.J.J. and T.L. conceived the study. J.J.J. analysed the data and wrote the first manuscript draft. J.J., A.P., K.A.‐S., J.I.H., J.E., P.S., J.L., S.M. and T.L. contributed to the concept and design of the study, interpretation of the results, and manuscript revision. All authors approved the final version of the manuscript. J.J.J. is responsible for the overall content as guarantor.

## Funding

The authors disclosed a receipt of the following financial support for the research, authorship and/or publication of this article: J.J.J. and T.L. were supported by the Research Council of Finland, Strategic Research Council (#358457). A.P. was supported by the European Union under grant agreement #101057739 (the TRIGGER project). J.I.H. was supported by the Research Council of Finland, Strategic Research Council (#358454). J.E. was supported by the Research Council of Finland, Strategic Research Council (#358458) and the Finnish Work Environment Fund (#220245). P.S. was supported by the Research Council of Finland, Strategic Research Council (#335186). S.M. was supported by the Research Council of Finland competitive funding to strengthen university research profiles (PROFI) for the University of Eastern Finland (#325022; #352968).

## Ethics Statement

The data collection of the FinHealth 2017 Study was approved by the Coordinating Ethics Committee at the Hospital District of Helsinki and Uusimaa (37/13/03/00/2016). Participants gave informed consent to participate in the FinHealth 2017 Study.

## Consent

The authors have nothing to report.

## Conflicts of Interest

The authors declare no conflicts of interest.

## Permission to Reproduce Material From Other Sources

The authors have nothing to report.

## Supporting information


Supporting Information S1

## Data Availability

The data of the FinHealth 2017 Study are available for research purposes in collaboration with the project organisation and through THL Biobank. In order to obtain access to the data, researchers must first submit a study proposal to the FinHealth 2017 or the THL Biobank Scientific Board. The forms to apply access to the data are available on the website of the FinHealth 2017 Study and the THL Biobank. The FinHealth 2017 website includes all the forms used during the fieldwork and the corresponding variables. Once access to the data is granted by the Scientific Board, a signed agreement is required, and all researchers must confirm to follow the THL data security rules.
