# Supplementary material for: Associations of Domain‐Specific Physical Activity With Mental Health Symptoms Among Finnish Employed Adults: A Population‐Based Study
Source: Eur J Sport Sci. 2026 Jan 7;26(2):e70118. doi: 10.1002/ejsc.70118 (PMC12777544; doi:10.1002/ejsc.70118)
Supplement: Supplementary file 1 — Supporting Information S1 [file EJSC-26-e70118-s001.docx]

# **SUPPLEMENTARY MATERIAL**

**Table S1.** Characteristics of the analytical sample by mental health symptom groups. Values are numbers (percentages).

| **Characteristics** | **No depressive symptoms**  ***N = 2677*** | **Depressive symptoms**  ***N = 762*** | **No psychological distress**  ***N = 3213*** | **Psychological distress**  ***N = 226*** |
| --- | --- | --- | --- | --- |
| **Sociodemographic** |  |  |  |  |
| Age |  |  |  |  |
| *18-34-years-old* | 618 (23.1) | 239 (31.4) | 787 (24.5) | 70 (31.0) |
| *35-54-years old* | 1355 (50.6) | 373 (48.9) | 1620 (50.4) | 108 (47.8) |
| *55-74-years old* | 704 (26.3) | 150 (19.7) | 806 (25.1) | 48 (21.2) |
| Sex |  |  |  |  |
| *Female* | 1322 (49.4) | 448 (58.8) | 1637 (50.9) | 133 (58.8) |
| *Male* | 1355 (50.6) | 314 (41.2) | 1576 (49.1) | 93 (41.2) |
| Education level |  |  |  |  |
| *Primary education* | 218 (8.1) | 57 (7.5) | 253 (7.9) | 22 (9.7) |
| *Secondary education* | 931 (34.8) | 304 (39.9) | 1141 (35.5) | 94 (41.6) |
| *Higher education* | 1528 (57.1) | 401 (52.6) | 1819 (56.6) | 110 (48.7) |
| Household income |  |  |  |  |
| *Low income* | 313 (11.7) | 163 (21.4) | 419 (13.0) | 57 (25.2) |
| *Middle income* | 1714 (64.0) | 491 (64.4) | 2061 (64.2) | 144 (63.7) |
| *High income* | 650 (24.3) | 108 (14.2) | 733 (22.8) | 25 (11.1) |
| Income satisfaction |  |  |  |  |
| *Unsatisfied* | 200 (7.5) | 186 (24.4) | 303 (9.4) | 83 (36.7) |
| *Satisfied* | 2475 (92.5) | 575 (75.6) | 2907 (90.6) | 143 (63.3) |
| Marital status |  |  |  |  |
| *Unmarried, divorced, or widowed* | 624 (23.3) | 264 (34.6) | 803 (25.0) | 85 (37.6) |
| *Married or cohabiting* | 2053 (76.7) | 498 (65.3) | 2410 (75.0) | 141 (62.4) |
| Children (living at home) |  |  |  |  |
| *No* | 1619 (60.5) | 493 (64.7) | 1956 (60.9) | 156 (69.0) |
| *Yes, at least one* | 1058 (39.5) | 269 (35.3) | 1257 (39.1) | 70 (31.0) |
| **Health status** |  |  |  |  |
| Body mass index |  |  |  |  |
| *Normal weight or underweight* | 1159 (43.3) | 311 (40.8) | 1388 (43.2) | 82 (36.3) |
| *Overweight or obese* | 1518 (56.7) | 451 (59.2) | 1825 (56.8) | 144 (63.7) |
| Self-rated health |  |  |  |  |
| *Poor or quite poor* | 40 (1.5) | 64 (8.4) | 69 (2.1) | 35 (15.5) |
| *Average* | 431 (16.1) | 259 (34.0) | 599 (18.7) | 91 (40.3) |
| *Good or quite good* | 2199 (82.4) | 438 (57.6) | 2537 (79.2) | 100 (44.2) |
| Chronic diseases |  |  |  |  |
| *No* | 1358 (52.5) | 317 (43.7) | 1592 (51.4) | 83 (39.0) |
| *Yes, at least one* | 1227 (47.5) | 409 (56.3) | 1506 (48.6) | 130 (61.0) |
| **Health behaviour** |  |  |  |  |
| Smoking status |  |  |  |  |
| *Non-smoker* | 1619 (60.5) | 365 (47.9) | 1893 (58.9) | 91 (40.3) |
| *Former smoker* | 635 (23.7) | 197 (25.9) | 760 (23.7) | 72 (31.8) |
| *Smoker* | 423 (15.8) | 200 (26.2) | 560 (17.4) | 63 (27.9) |
| Hazardous or problem drinking |  |  |  |  |
| *No* | 2008 (75.0) | 519 (68.1) | 2383 (74.2) | 144 (63.7) |
| *Yes* | 669 (25.0) | 243 (31.9) | 830 (25.8) | 82 (36.3) |
| Adequate sleep |  |  |  |  |
| *No, seldom or never or cannot tell* | 452 (20.7) | 305 (40.3) | 735 (23.0) | 122 (54.7) |
| *Yes, almost always or often* | 2113 (79.3) | 552 (59.7) | 2464 (77.0) | 101 (45.3) |

All variables globally significantly different between mental health symptom groups at *p* < 0.05, except age (psychological distress 0.078), education (psychological distress 0.065), and body mass index (depression symptoms 0.222).

**Table S2.** Adjusted odds ratios with 95% confidence intervals for mental health symptoms by commuting profile and active commuting volume.

|  | **Model 1** | **Model 2** | **Model 3 (Main)** | **Model 4** | **Model 5** | **Model 6** | **Model 7** | **Model 8** |
| --- | --- | --- | --- | --- | --- | --- | --- | --- |
| **Depressive symptoms (profile)** |  |  |  |  |  |  |  |  |
| Passive commuters | 1 | 1 | 1 | 1 | 1 | 1 | 1 | 1 |
| Active commuters | 1.35 (1.10-1.66) | 1.23 (1.00-1.52) | 1.20 (0.97-1.48) | 1.21 (0.98-1.50) | 1.17 (0.95-1.45) | 1.24 (1.00-1.55) | 1.24 (0.99-1.56) | 1.28 (1.03-1.58) |
| **Depressive symptoms (volume)** |  |  |  |  |  |  |  |  |
| Passive commuting | 1 | 1 | 1 | 1 | 1 | 1 | 1 | 1 |
| < 15 minutes a day | 0.92 (0.68-1.24) | 0.90 (0.64-1.26) | 0.84 (0.59-1.20) | 0.85 (0.59-1.21) | 0.82 (0.57-1.18) | 0.85 (0.59-1.22) | 0.89 (0.62-1.28) | 0.87 (0.62-1.23) |
| 15-29 minutes a day | 1.43 (1.03-1.98) | 1.25 (0.90-1.75) | 1.20 (0.87-1.65) | 1.21 (0.88-1.68) | 1.18 (0.85-1.63) | 1.27 (0.91-1.79) | 1.26 (0.90-1.76) | 1.29 (0.94-1.76) |
| ≥ 30 minutes a day | 1.70 (1.28-2.25) | 1.56 (1.16-2.10) | 1.58 (1.18-2.13) | 1.62 (1.21-2.17) | 1.56 (1.16-2.09) | 1.64 (1.20-2.24) | 1.59 (1.17-2.16) | 1.70 (1.26-2.28) |
| **Psychological distress (profile)** |  |  |  |  |  |  |  |  |
| Passive commuters | 1 | 1 | 1 | 1 | 1 | 1 | 1 | 1 |
| Active commuters | 1.41 (1.02-1.95) | 1.33 (0.95-1.87) | 1.31 (0.93-1.85) | 1.32 (0.94-1.85) | 1.23 (0.85-1.78) | 1.38 (0.99-1.93) | 1.30 (0.93-1.83) | 1.43 (1.03-1.97) |
| **Psychological distress (volume)** |  |  |  |  |  |  |  |  |
| Passive commuting | 1 | 1 | 1 | 1 | 1 | 1 | 1 | 1 |
| < 15 minutes a day | 1.26 (0.82-1.95) | 1.31 (0.82-2.07) | 1.24 (0.77-2.00) | 1.24 (0.77-2.00) | 1.15 (0.70-1.91) | 1.26 (0.77-2.07) | 1.26 (0.77-2.05) | 1.34 (0.85-2.12) |
| 15-29 minutes a day | 1.35 (0.89-2.07) | 1.24 (0.79-1.94) | 1.21 (0.78-1.89) | 1.22 (0.78-1.89) | 1.18 (0.74-1.89) | 1.32 (0.85-2.06) | 1.31 (0.83-2.05) | 1.29 (0.82-2.02) |
| ≥ 30 minutes a day | 1.62 (0.98-2.69) | 1.46 (0.85-2.49) | 1.49 (0.88-2.53) | 1.51 (0.90-2.53) | 1.36 (0.79-2.34) | 1.56 (0.92-2.64) | 1.34 (0.79-2.26) | 1.65 (0.99-2.76) |

*Model 1*. Adjusted for age and sex.

*Model 2*. Adjusted for age, sex, education level, household income, marital status, number of children (living at home), smoking status, hazardous or problem drinking.

*Model 3 (Main)*. Adjusted for age, sex, education level, household income, marital status, number of children (living at home), smoking status, hazardous or problem drinking, leisure-time physical activity, and occupational physical activity.

*Model 4*. Adjusted for age, sex, education level, household income, marital status, number of children (living at home), smoking status, hazardous or problem drinking, leisure-time physical activity, occupational physical activity, and body mass index.

*Model 5*. Adjusted for age, sex, education level, household income, marital status, number of children (living at home), smoking status, hazardous or problem drinking, leisure-time physical activity, occupational physical activity, and adequate sleep.

*Model 6*. Adjusted for age, sex, education level, household income, marital status, number of children (living at home), smoking status, hazardous or problem drinking, leisure-time physical activity, occupational physical activity, and self-rated health.

*Model 7*. Adjusted for age, sex, education level, household income, marital status, number of children (living at home), smoking status, hazardous or problem drinking, leisure-time physical activity, occupational physical activity, and history of chronic diseases.

*Model 8*. Adjusted for age, sex, education level, income satisfaction, marital status, number of children (living at home), smoking status, hazardous or problem drinking, leisure-time physical activity, and occupational physical activity.

**Table S3.** Adjusted odds ratios with 95% confidence intervals for mental health symptoms by occupational physical activity profile.

|  | **Model 1** | **Model 2** | **Model 3 (Main)** | **Model 4** | **Model 5** | **Model 6** | **Model 7** | **Model** 8 |
| --- | --- | --- | --- | --- | --- | --- | --- | --- |
| **Depressive symptoms** |  |  |  |  |  |  |  |  |
| Sedentary | 1 | 1 | 1 | 1 | 1 | 1 | 1 | 1 |
| Lightly active | 0.88 (0.66-1.17) | 0.79 (0.59-1.07) | 0.79 (0.58-1.06) | 0.79 (0.58-1.06) | 0.75 (0.55-1.03) | 0.82 (0.60-1.13) | 0.80 (0.58-1.08) | 0.83 (0.61-1.12) |
| Moderately or highly active | 1.08 (0.84-1.39) | 0.87 (0.65-1.16) | 0.88 (0.66-1.17) | 0.89 (0.67-1.18) | 0.86 (0.65-1.15) | 0.87 (0.65-1.18) | 0.85 (0.64-1.14) | 0.95 (0.72-1.25) |
| **Psychological distress** |  |  |  |  |  |  |  |  |
| Sedentary | 1 | 1 | 1 | 1 | 1 | 1 | 1 | 1 |
| Lightly active | 0.71 (0.47-1.08) | 0.64 (0.41-0.98) | 0.62 (0.40-0.97) | 0.62 (0.40-0.97) | 0.54 (0.33-0.88) | 0.70 (0.45-1.10) | 0.65 (0.41-1.03) | 0.69 (0.44-1.08) |
| Moderately or highly active | 1.18 (0.81-1.72) | 0.95 (0.59-1.51) | 0.97 (0.61-1.55) | 0.98 (0.61-1.55) | 0.89 (0.56-1.42) | 1.01 (0.61-1.67) | 0.94 (0.58-1.51) | 1.10 (0.69-1.77) |

*Model 1*. Adjusted for age and sex.

*Model 2*. Adjusted for age, sex, education level, household income, marital status, number of children (living at home), smoking status, hazardous or problem drinking.

*Model 3 (Main)*. Adjusted for age, sex, education level, household income, marital status, number of children (living at home), smoking status, hazardous or problem drinking, active commuting, and leisure-time physical activity.

*Model 4*. Adjusted for age, sex, education level, household income, marital status, number of children (living at home), smoking status, hazardous or problem drinking, active commuting, leisure-time physical activity, and body mass index.

*Model 5*. Adjusted for age, sex, education level, household income, marital status, number of children (living at home), smoking status, hazardous or problem drinking, active commuting, leisure-time physical activity, and adequate sleep.

*Model 6*. Adjusted for age, sex, education level, household income, marital status, number of children (living at home), smoking status, hazardous or problem drinking, active commuting, leisure-time physical activity, and self-rated health.

*Model 7*. Adjusted for age, sex, education level, household income, marital status, number of children (living at home), smoking status, hazardous or problem drinking, active commuting, leisure-time physical activity, and history of main chronic diseases.

*Model 8*. Adjusted for age, sex, education level, income satisfaction, marital status, number of children (living at home), smoking status, hazardous or problem drinking, active commuting, and leisure-time physical activity.

**Table S4.** Adjusted odds ratios with 95% confidence intervals for mental health symptoms by leisure-time physical activity profile.

|  | **Model 1** | **Model 2** | **Model 3 (Main)** | **Model 4** | **Model 5** | **Model 6** | **Model 7** | **Model** 8 |
| --- | --- | --- | --- | --- | --- | --- | --- | --- |
| **Depressive symptoms** |  |  |  |  |  |  |  |  |
| Sedentary | 1 | 1 | 1 | 1 | 1 | 1 | 1 | 1 |
| Recreationally active | 0.54 (0.42-0.68) | 0.56 (0.44-0.71) | 0.57 (0.44-0.73) | 0.58 (0.46-0.75) | 0.62 (0.49-0.79) | 0.66 (0.51-0.87) | 0.56 (0.43-0.73) | 0.59 (0.46-0.76) |
| Exercisers and athletes | 0.39 (0.29-0.54) | 0.45 (0.32-0.62) | 0.44 (0.32-0.61) | 0.46 (0.33-0.63) | 0.51 (0.37-0.70) | 0.60 (0.42-0.86) | 0.45 (0.32-0.62) | 0.43 (0.31-0.60) |
| **Psychological distress** |  |  |  |  |  |  |  |  |
| Sedentary | 1 | 1 | 1 | 1 | 1 | 1 | 1 | 1 |
| Recreationally active | 0.44 (0.30-0.63) | 0.46 (0.31-0.67) | 0.46 (0.32-0.68) | 0.47 (0.32-0.68) | 0.51 (0.34-0.76) | 0.62 (0.41-0.93) | 0.43 (0.29-0.63) | 0.49 (0.34-0.72) |
| Exercisers and athletes | 0.30 (0.18-0.46) | 0.35 (0.22-0.57) | 0.34 (0.21-0.55) | 0.35 (0.22-0.56) | 0.41 (0.25-0.68) | 0.63 (0.36-1.08) | 0.37 (0.23-0.60) | 0.35 (0.21-0.58) |

*Model 1*. Adjusted for age and sex.

*Model 2*. Adjusted for age, sex, education level, household income, marital status, number of children (living at home), smoking status, hazardous or problem drinking.

*Model 3 (Main)*. Adjusted for age, sex, education level, household income, marital status, number of children (living at home), smoking status, hazardous or problem drinking, active commuting, and occupational physical activity.

*Model 4*. Adjusted for age, sex, education level, household income, marital status, number of children (living at home), smoking status, hazardous or problem drinking, active commuting, occupational physical activity, and body mass index.

*Model 5*. Adjusted for age, sex, education level, household income, marital status, number of children (living at home), smoking status, hazardous or problem drinking, active commuting, occupational physical activity, and adequate sleep.

*Model 6*. Adjusted for age, sex, education level, household income, marital status, number of children (living at home), smoking status, hazardous or problem drinking, active commuting, occupational physical activity, and self-rated health.

*Model 7*. Adjusted for age, sex, education level, household income, marital status, number of children (living at home), smoking status, hazardous or problem drinking, active commuting, occupational physical activity, and history of chronic diseases.

*Model 8*. Adjusted for age, sex, education level, income satisfaction, marital status, number of children (living at home), smoking status, hazardous or problem drinking, active commuting, and occupational physical activity.
